# Performance of Repetitive Tasks Induces Decreased Grip Strength and Increased Fibrogenic Proteins in Skeletal Muscle: Role of Force and Inflammation

**DOI:** 10.1371/journal.pone.0038359

**Published:** 2012-05-31

**Authors:** Samir M. Abdelmagid, Ann E. Barr, Mario Rico, Mamta Amin, Judith Litvin, Steven N. Popoff, Fayez F. Safadi, Mary F. Barbe

**Affiliations:** 1 Department of Surgery, Plastic and Reconstructive Division, Children's Hospital of Philadelphia, Philadelphia, Pennsylvania, United States of America; 2 College of Health Professions, Pacific University, Hillsboro, Oregon, United States of America; 3 Sol Sherry Thrombosis Research Center, Temple University School of Medicine, Philadelphia, Pennsylvania, United States of America; 4 Department of Anatomy and Cell Biology, Temple University School of Medicine, Philadelphia, Pennsylvania, United States of America; 5 Musculoskeletal Research Group, Temple University School of Medicine, Philadelphia, Pennsylvania, United States of America; 6 Department of Anatomy and Neurobiology, Northeast Ohio Medical University (NEOMED), Rootstown, Ohio, United States of America; Northwestern University Feinberg School of Medicine, United States of America

## Abstract

**Background:**

This study elucidates exposure-response relationships between performance of repetitive tasks, grip strength declines, and fibrogenic-related protein changes in muscles, and their link to inflammation. Specifically, we examined forearm flexor digitorum muscles for changes in connective tissue growth factor (CTGF; a matrix protein associated with fibrosis), collagen type I (Col1; a matrix component), and transforming growth factor beta 1 (TGFB1; an upstream modulator of CTGF and collagen), in rats performing one of two repetitive tasks, with or without anti-inflammatory drugs.

**Methodology/Results:**

To examine the roles of force versus repetition, rats performed either a high repetition negligible force food retrieval task (HRNF), or a high repetition high force handle-pulling task (HRHF), for up to 9 weeks, with results compared to trained only (TR-NF or TR-HF) and normal control rats. Grip strength declined with both tasks, with the greatest declines in 9-week HRHF rats. Quantitative PCR (qPCR) analyses of HRNF muscles showed increased expression of Col1 in weeks 3–9, and CTGF in weeks 6 and 9. Immunohistochemistry confirmed PCR results, and also showed greater increases of CTGF and collagen matrix in 9-week HRHF rats than 9-week HRNF rats. ELISA, and immunohistochemistry revealed greater increases of TGFB1 in TR-HF and 6-week HRHF, compared to 6-week HRNF rats. To examine the role of inflammation, results from 6-week HRHF rats were compared to rats receiving ibuprofen or anti-TNF-α treatment in HRHF weeks 4–6. Both treatments attenuated HRHF-induced increases in CTGF and fibrosis by 6 weeks of task performance. Ibuprofen attenuated TGFB1 increases and grip strength declines, matching our prior results with anti-TNFα.

**Conclusions/Significance:**

Performance of highly repetitive tasks was associated with force-dependent declines in grip strength and increased fibrogenic-related proteins in flexor digitorum muscles. These changes were attenuated, at least short-term, by anti-inflammatory treatments.

## Introduction

Work-related musculoskeletal disorders (WMSDs) occurring as a result of repeated trauma continue to cause substantial numbers of lost work days [1], disability and discomfort in US industry. WMSDs include several diagnoses, including muscle disorders [2], [3]. However, the mechanisms leading to pathophysiological tissue changes associated with WMSDs are incompletely understood. The 2010 National Manufacturing Agenda (NORA) of the National Institute of Occupational Safety and Health (NIOSH) cites the need for etiologic research in determining the contribution of biomechanical mechanisms (e.g., repetitive motion) towards the development of tissue injury and musculoskeletal disorders, as well as strategies to reduce their severity [4].

Several animal models have been developed to study WMSDs and have shown that repetitive hand activities induce motor dysfunction [5], [6], [7], [8], [9], [10], [11], [12], [13], [14], [15]. When examining the effects of performing either a high repetition or low repetition food retrieval task (a negligible force task) in our rat model of voluntary repetitive working, we observed exposure-dependent declines in grip strength, with greater declines with the high repetition task [6], [11], [16]. Performance of a high repetition, high force (HRHF) handle-pulling task induced even greater declines in grip strength [7], [12], [17]. However, mechanisms underlying these declines in motor strength are still under investigation.

Some mechanisms examined to date in our model include task-induced tissue injury, inflammation and fibrosis, each of which may contribute to declines in grip strength by producing discomfort or affecting biomechanical strength. Evidence of injury includes focal myofiber fray as well as compression and degraded myelin in the median nerve with performance of the high repetition, negligible force (HRNF) task for 8 weeks [5]. Greater signs of tissue injury were observed with performance of higher force tasks, including moth-eaten muscle fibers [18]. These injuries were paralleled by inflammatory responses, such as increased tissue pro-inflammatory cytokines and macrophages [5], [7], [8], [19], [20]. These increases in inflammatory responses preceded fibrotic responses in forelimb nerves, tendons and periosteum, including connective tissue hyperplasia and increased connective tissue growth factor (CTGF), periostin, periostin like factor (PLF), and collagen type 1 [7], [8], [12], [17], [18], [21]. However, we have yet to examine muscles for similar fibrotic responses to these repetitive tasks. If present, muscle fibrosis may well alter muscle biomechanics in a manner that reduces grip strength [22], [23], [24].

Muscles have been shown to undergo repetitive strain-induced fibrotic changes in forced lengthening models of repetitive motion. Stauber and colleagues have shown that repeated muscle strains at fast velocities (lengthening contractions stimulated electrically) resulted in fibrotic myopathy with increased collagen content, collagen cross-links and non-contractile tissues, while repeated strains at low velocities did not [25], [26], [27], [28]. Factors and mechanisms of repetitive strain-induced fibrosis in tissues are still under investigation, but appear to involve CTGF and transforming growth factor beta-1 (TGFB1) [29], [30], [31], [32], [33]. Unfortunately, recovery from fibrotic changes is slow, even with complete cessation of strain/activity for up to 3 months [27]. Blocking tumor necrosis factor alpha (TNF-α) with antibodies has been used to successfully reduce inflammation-induced kidney and liver fibrosis, as well as collagen-induced arthritis, with subsequent improvement in symptoms [34], [35], [36]. We have recently shown that anti-rat TNF-α during the early stages of inflammation attenuates repetitive task-induced increases of PLF, a matricellular molecule that increases with tissue loading in musculoskeletal tissues, and that TNF-α regulates PLF production in osteoblasts *in vitro* [12]. Production of CTGF by fibroblasts, another matricellular protein, is also regulated by TNF-α [37], [38], although the regulation of CTGF by TNF-α in muscles undergoing repeated strain has yet to be investigated. The link between these matricellular proteins and TNF-α gives insight into possible mechanisms (i.e. inflammatory) that may drive fibrotic tissue repair.

Our aims were to determine: a) if fibrogenic-related proteins increase in skeletal muscles in response to prolonged repetitive reaching, b) if their increases parallel declines in grip strength, c) the extent to which increased force loads contribute to either change, and d) if anti-inflammatory treatments attenuate these responses. Since CTGF, collagen type 1, and TGFB1 increase under conditions of muscle overload or injury and are linked to tissue fibrosis [7], [8], [17], [29], [30], [39], [40], [41], we hypothesized that these proteins would increase with continued task performance and/or force loads. Since these proteins are linked to tissue fibrosis, we hypothesized that their increases would parallel grip strength declines. We further hypothesized that, as with PLF [12], [18], [21], that two other matrix regulatory proteins, TGFB1 and CTGF, may also be linked to an early task-induced inflammatory response. If so, then early administration of anti-inflammatory drugs should decrease their production, the onset of muscle fibrosis, and attenuate task-induced grip strength declines.

## Methods

### Animals

All experiments were approved by the Temple University Institutional Animal Care and Use Committee (Temple University IACUC) in compliance with NIH guidelines for the humane care and use of laboratory animals. Rats were housed individually in the central animal facility in transparent plastic cages in a 12 hour light: 12 hour dark cycle with free access to water. Studies were conducted on a total of 142, young adult (3.5–4 months of age at onset of experiments), female, Sprague-Dawley rats.

### Overview of training and task regimens

Seventeen rats served as age-matched normal controls (NC) with free access to food, and did not undergo training or task performance; 9 more NC rats received ibuprofen treatment (NC+IBU). All but NC and NC+IBU rats were food restricted (n = 117) to within 5% of their naïve weights as described previously [9], [16]. These food restricted rats went through an initial training period in which they learned to perform either a high repetition negligible force, food retrieval task (TR-NF, n = 51) as described previously [5], or a high repetition high force, handle-pulling task (TR-HF, n = 56) as described previously [7], [18], [21], for a food reward. The rats were trained for 10 min/day, 5 days/week. This training period was either 10 days (TR-NF) or 5–6 weeks (TR-HF) (learning to perform the high force task took longer than the negligible force task).

Forty-one of the TR-NF rats went on to perform the high repetition negligible force, food retrieval task (HRNF). These 41 rats performed the HRNF task for 2 hr/day, 3 days/week for 3 weeks (n = 12), 6 weeks (n = 11) or 9 weeks (n = 18). Thirty-four of the TR-HF rats went on to perform the high repetition high force, handle-pulling task (HRHF). These 34 rats performed the HRHF task for 2 hr/day, 3 days/week, for either 6 weeks (n = 29), or 9 weeks (n = 5). Subcohorts of the 6-week HRHF rats either went untreated (6 HRHF, n = 11), or were treated with anti-rat TNF-α (6HRHF+anti-TNF, n = 6) or ibuprofen (6HRHF+IBU, n = 12) in weeks 5 and 6 of HRHF task performance. The remaining TR-NF (n = 10) or TR-HF rats (n = 32) were euthanized after the training period, and served as trained-only controls. Subcohorts of TR-HF rats, either went untreated (TR-HF, n = 17), or received anti-rat TNF-α (TR-HF+anti-TNF, n = 6) or ibuprofen (TR-HF+IBU, n = 9) before euthanasia, as described further below.

All rats were weighed weekly and food adjusted accordingly. In addition to food pellet rewards, all rats received Purina rat chow daily. Trained-only rats received food pellets and rat chow that matched allotments given to task rats. In accordance with the recommendations of the Panel on Euthanasia of the American Veterinary Medical Association, animals were euthanized using sodium pentobarbital (120 mg/kg body weight) before tissue collection.

### Description of HRNF and HRHF task regimens

The HRNF and HRHF tasks have been described in detail previously [5], [7], [18], [21]. Briefly, the HRNF task was a forelimb food pellet retrieval task in which rats reached and retrieved a food pellet at a target reach rate of 4 reaches/min and <5% maximum pulling force (MPF; the 45 mg food pellet was estimated as <5% MPF) in customized operant behavioral apparati (Med Associates, St Albans, VT), as described previously [5], [6]. Force-dependent changes were examined by having additional rats perform a high repetition high force task (HRHF) at a target rate of 4 reaches/min and 60% maximum pulling force. This task was a forelimb handle-pulling task for a food reward that used customized operant behavioral chambers as described previously [7], [17], [18].

### Anti-inflammatory treatments

*Ibuprofen:* At the end of the 4th week of HRHF task performance, 12 of the HRHF rats were administered liquid ibuprofen (Children's Motrin Grape Flavored, Johnson & Johnson, New Brunswick, New Jersey) in drinking water daily (45 mg/kg body weight), as described previously [42]. These animals continued to perform the HRHF task regimen for two more weeks while receiving the ibuprofen treatment (6HRHF+IBU). Nine of the TR-HF rats (TR-HF+IBU) and 9 normal controls (NC+IBU) also received ibuprofen for 2 weeks. The ibuprofen dose used was moderate, yet effective in reducing HRHF-induced increases in tissue cyclooxygenase 2 and pro-inflammatory cytokines in our rat model [42]. The amount of medicated water consumed/day was tracked for each animal by measuring the difference between the initial and final volume of suspended solution daily. Blood was collected, centrifuged, and serum levels of ibuprofen were tested using National Medical Services (NMS, Willow Grove, PA). By these measures, it was determined that all ibuprofen treated rats had between 48.8±6.3 mg/kg body weight of ibuprofen daily.

*Anti-TNF-α:* Another subcohort of HRHF rats were administered an anti-rat TNF-α (CNTO 1081; generously provided by Janssen R&D), as described previously [12]. Briefly, this drug was injected intraperitoneally (i.p., 15 mg/kg body wt.) into HRHF rats beginning mid-task week 4, at the end of task week 4, and at the end of task week 5 (6HRHF+anti-TNF, n = 6). Six of the TR-HF rats received similar anti-TNFα treatment (TR-HF+anti-TNF).

### Determination of daily exposure and grip strength

Daily exposure (reaches/day) was obtained from: (a) 22 randomly chosen HRNF animals at the end of task weeks 1 and 3 (n = 22 each), and weeks 6 and 9 (n = 18 each) of HRNF task performance [the number reduces across weeks due to euthanasia for tissue analysis]; and (b) 14 randomly chosen untreated HRHF animals at the end of task weeks 1 and 3 (n = 14 each), and weeks 6 and 9 (n = 7 each) of HRHF task performance. The mean daily exposure, reaches/day, was determined by multiplying the reach rate by the number of minutes per day that the rats participated in the task.

Average grip strength was tested using previously described methods [6] in: (a) 24 randomly chosen HRNF animals at the naïve time point, at the end of training (week 0; TR), and in weeks 1–5, and weeks 6–9 (n = 18 each) of HRNF task performance; and from (b) 38 randomly chosen untreated HRHF animals at the naïve time point, at the end of training (week 0; TR), and in weeks 1–4, weeks 5 and 6 (n = 35), and weeks 7–9 (n = 9) of HRHF task performance. Average grip strength was also determined in NC+IBU (n = 9), TR-HF+IBU (n = 9), and 6HRHF+IBU rats (n = 12). Grip strength has already been reported for anti-TNF-α treated rats [12].

### Quantitative Real-Time PCR (qPCR)

**Figure 1:**
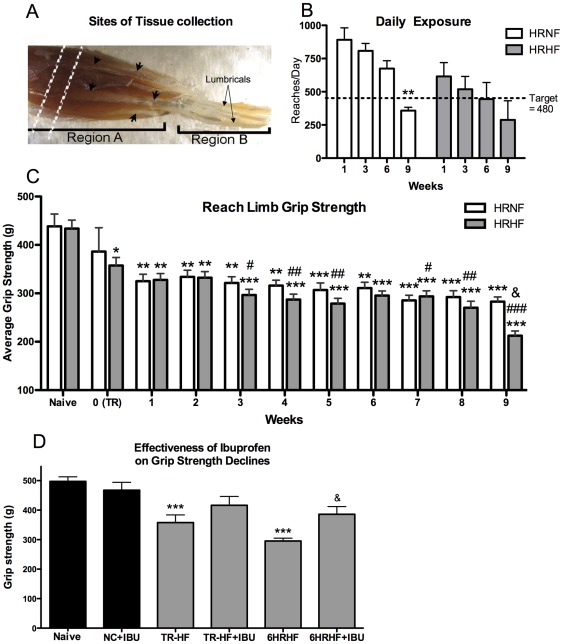
Forelimb tissues examined, daily exposure, and grip strength declines. *(A)* Flexor digitorum muscles were collected from region A of the flexor digitorum muscles, a region containing primarily muscle but also proximal tendon slips (short arrows). The vertical dashed lines indicate the region of muscle cut into cross-section for histological analysis. The remaining part of region A and region B were sectioned together longitudinally. Lumbricals are located in region B (long arrows in the palm). Region A was used for PCR, ELISA and western blot analyses in separate animals than those for histology. *(B)* Daily exposure (mean reaches/day) decreased similarly in both groups with continued task performance, and dropped below the target of 480 reaches/day (indicated by dotted line) in each by week 9, although significantly lower only in week 9 HRNF rats compared to their week 1 level. **p<0.01 compared to same group week 1. *(C)* Average grip strength decreased in both groups with continued task performance, with the greatest declines in HRHF week 9. *p<0.05, **p<0.01, ***p<0.001, compared to naïve; ^#^p<0.05, ^##^p<0.01, ^###^p<0.001 compared to HRHF week 0 (end of training period (TR) for TR-HF rats); ^&^p<0.05 compared to 9-week HRNF rats. *(D)* Grip strength declines in the preferred reach limb of trained (TR-HF) and 6-week HRHF rats were attenuated after two weeks of ibuprofen treatment in trained rats (TR-HF+IBU) and HRHF rats (6HRHF+IBU). ***p<0.001 compared to NC; ^&^p<0.05 compared to untreated 6-week HRHF rats.

Fifteen animals were euthanized with an overdose of sodium pentobarbital (120 mg/kg body weight) and forearm flexor muscles (and associated proximal tendon slips) were collected from Region A of the flexor digitorum mass as shown in Figure 1A from NC, TR-NF, and 3-, 6- and 9-week HRNF rats (n = 3/group). Each muscle belly was divided into two longitudinal parts; half was used for RNA extraction and half for protein extraction. The half for RNA extraction was put into RNAlater RNA Stabilization Reagent (QIAGEN, Valencia, CA) for 2 hours at room temperature, and then stored at −80°C. Total RNA was isolated using TRIzol reagent (Invitrogen, Carlsbad, CA). The concentration of each RNA sample was determined using a spectrophotometer; the integrity was monitored on 1% formaldehyde denatured gels. After confirming RNA integrity, cDNA was prepared from mRNA extracts from the above tissue samples (n = 3/group), using a High Capacity cDNA Reverse Transcription kit (Applied Biosystems™, Foster City, CA). PCR primer sets for the following were used: Collagen type I alpha1 (Col1; Cat# PP42922APPR42922A), CTGF (Cat# PPR46426A) and GAPDH (Cat# PPR51520A) (SABiosciences, Frederick, MD). qPCR was then performed in duplicate for 20 µl reactions, using the SYBR Green PCR Master Mix method (Applied Biosystems, Foster City, CA) on an ABI 7500 Fast Real-Time PCR system (Applied Biosystems™). PCR cycles consisted of an initial cycle of 50°C for 2 min and the second cycle of 95°C for 10 min, followed by a two-step program of 95°C for 15 sec and 60°C for 1 min for 40 cycles. Using GAPDH as the internal control, relative gene expression among samples was calculated by comparison of C_t_ (threshold cycle) values. A dissociation curve was checked for each qPCR run to confirm specific amplification of target RNA.

### Synthesis of CTGF riboprobe and in situ hybridization

A CTGF cDNA fragment of approximately 700 bp of the 3^′^ end of the rat CTGF cDNA was cloned into PCR-Script vector (Stratagene, LaJolla, CA) flanked by the T3 and T7 promoters to generate CTGF sense and CTGF anti-sense riboprobes. Concentration of the sense and anti-sense riboprobe was determined using an in situ hybridization labeling kit (Roche Diagnostic). For in situ hybridization of CTGF in flexor digitorum muscles, animals were euthanized and flexor digitorum muscles and tendons from NC and 9-week HRNF rats were harvested (n = 3/group), fixed in 4% paraformaldehyde in phosphate buffered saline (PBS) at 4°C, dehydrated, embedded in paraffin, sectioned (5 µm sections), and placed on charged slides (Super Frost Plus, ThermoFisher Scientific, Pittsburgh, PA). In situ hybridization was then carried out as described previously [43].

### Immunohistochemical analyses

Animals received an overdose of sodium pentobarbital (120 mg/kg body weight) before being perfused transcardially with 4% paraformaldehyde in 0.1 M phosphate buffer (pH 7.4): NC (n = 6); TR-NF (n = 5); TR-HF (n = 10), TR-HF+IBU (n = 4), TR-HF+anti-TNF (n = 3); 3-week HRNF (n = 7); 6-week HRNF (n = 5); 9-week HRNF (n = 10); 6-week HRHF (n = 6); 9-week HRHF (n = 4); 6HRHF+IBU (n = 7); and 6HRHF+anti-TNF (n = 3). Tissues were collected and postfixed “en bloc” by immersion overnight. A proximal portion of the muscle mass was then removed with a scalpel for cross-sectional sectioning (as shown with vertical dashed lines in Fig. 1A), while the remaining flexor digitorum muscles and tendons were separated *en bloc* as a flexor mass from the bones for longitudinal sectioning (included both Regions A and B as shown in Fig. 1A). All tissues were cryoprotected in 30% sucrose in PBS before frozen-sectioning using a cryostat into 15 µm longitudinal or cross sectional slices. Sections were then placed onto charged slides (Fisher, Super Frost Plus) and allowed to dry overnight before storage at −80°C.

Sections on slides were treated with 3% H_2_O_2_ in methanol to block for endogenous peroxidase for 30 min, washed in PBS, and then blocked with 10% goat serum in PBS for 20 min at room temperature. For localization of OA, sections were first permeabilized with 0.05% pepsin in 0.01N HCL, prior to blocking with 10% goat serum in PBS for 20 min, and prior to primary antibody incubation. Sections were then incubated overnight at room temperature with primary antibody diluted in 10% goat serum in PBS. Primary antibodies were as follows: anti-CTGF (Cat# sc-14939, 1∶400 dilution, Santa Cruz Biotechnology, Santa Cruz, CA), anti-collagen type I (Cat# C2456, 1∶500 dilution, Sigma-Aldrich, St. Louis, MO), and TGFB1 (Cat# MAB240, 1∶300 dilution, R&D Systems). On the 2^nd^ day, after washing, sections were incubated with the appropriate secondary antibody conjugated to HRP (Jackson Immunoresearch Laboratories, West Grove, PA; each were diluted 1∶100 in PBS, and incubated 2 hours at room temperature before washing in PBS) and visualized using DAB with or without cobalt (black versus brown, respectively; Fast DAB, Sigma), or with appropriate secondary antibody conjugated to Cy2 (green fluorescent tag) or Cy3 (red fluorescent tag) (Jackson Immunoresearch Laboratories). DAB-treated sections were counterstained lightly with eosin. Slides were either dehydrated and coverslipped with DPX mounting medium for bright field microscopy (for HRP-DAB) or washed with PBS and coverslipped with 80% glycerol in PBS for epifluorescence microscopy. Negative control staining was performed by omitting either the primary antibody or the secondary antibody. Specificity of the TGFB1 antibody was determined via the use of a TGFB1 blocking (Santa Cruz Biotechnology Cat# sc-146 P). Western blot analysis, using methods described previously [12], was used to show that proteins of the correct molecular weight were immunodetected with the CTGF and collagen type I antibodies in skeletal muscle (as shown in the results section and figures). Quantification of the immunohistochemical findings was performed using an image analysis program (Bioquant, Nashville, TN) and methods, as described previously [17], [19]. Other subsets underwent Masson's trichrome staining as described [44].

### ELISA of musculoskeletal tissues

Animals were euthanized with an overdose of sodium pentobarbital (120 mg/kg body weight) before tissue collection from Region A of the flexor digitorum mass (Fig. 1A) from NC (n = 8), NC+IBU (n = 5), TR-NF (n = 5), TR-HF (n = 5), TR-HF+IBU (n = 5), TR-HF+anti-TNF (n = 3), 6-week HRNF (n = 6), 6-week HRHF (n = 5), 6HRHF+IBU (n = 5), and 6HRHF+anti-TNF (n = 3). As described above, the muscles were divided into half longitudinally. The half for protein analysis was snap frozen and stored at −80°C until homogenization, and then prepared for protein analysis, as described previously [6], [12]. Total protein was determined using BCA-200 protein assays (Bicinchoninic Acid, Pierce, Rockford, IL). For ELISA, tissue lysates (50 microliter aliquots) were analyzed using a commercially available ELISA kit for TGFB1 (Cat# PA1-9574, Pierce), according to the manufacturers' protocol. Each sample was run in duplicate. ELISA assay data (pg protein) were normalized to µg total protein.

### Statistical Analysis

Repeated measures, mixed model, two-way ANOVAs were used to analyze grip strength, with the factors group (HRNF and HRHF) and week (naïve, 0–9). Two-way ANOVAs, with the factors group and week, were used to analyze daily exposure and CTGF. Univariate ANOVA was used to analyze quantitative PCR and TGFB1 ELISA for differences in task weeks versus controls. Univariate ANOVA was also used to examine for drug effects on CTGF, TGFB1 and grip strength, to examine for any significant changes between controls, task and treatment groups. Statistical analyses were performed using Prism 5 (GraphPad Software, La Jolla, CA). All post hoc analyses were carried out using the Bonferroni test for multiple comparisons as indicated; adjusted p values are reported. An adjusted p value of <0.05 was considered significant for all analyses. Data are presented as the mean ± standard error of the mean (SEM).

## Results

### Daily exposure to repetitive tasks

Daily exposure showed significant declines towards the target of 480 reaches/day with continued task performance (Fig. 1B; 2-way ANOVA: week, p = 0.003; group, p = 0.01), suggestive of skill acquisition, decreased performance abilities, or a combination of the two. Specifically, in both groups, rats tended to overreach initially. By week 9, reaches/day declined below the target in HRNF rats, compared to HRNF week 1 (p<0.01), suggesting skill acquisition because it declined toward their target reach rate. In HRHF rats, reaches/day declined by week 9, but not significantly, compared to HRHF week 1, suggesting that rats in this higher force group are self-regulating their reach rate due to discomfort (as shown in Fedorczyk et al, 2010). Nevertheless, since reaches/day were similar statistically between the groups, force level rather than reaches/day was a key difference between the two groups (<5% maximum pulling force in HRNF compared to 60% in HRHF tasks rats).

### Grip Strength declined with continued performance of both tasks, more with HRHF

Grip strength showed significant declines with continued task performance in both groups (Fig. 1C; 2-way ANOVA: week, p<0.001; group, p = 0.03). Specifically, TR-NF rats did not have declines in grip strength compared to naïve rats (indicated as 0(TR) in Fig. 1C), but TR-HF rats showed significant declines by the end of their training period, compared to naïve rats (p<0.01). Each group showed declines in grip strength with continued task performance, compared to naïve rats (See Fig. 1C for p values). Compared across groups, 9-week HRHF rats had the greatest grip strength declines, compared to 9-week HRNF rats (p<0.05; Fig. 1C). Thus, both force level and chronicity of exposure contributed to declines in grip strength.

### Grip strength declines were attenuated by ibuprofen treatment

Since we have shown that administration of the anti-rat TNF-α during the early stages of inflammation attenuates HRHF task-induced declines in grip strength [12], we sought to determine here if ibuprofen treatment had a similar effect. Grip strength showed significant declines in each group with continued task performance (Fig. 1D; 1-way ANOVA: p<0.001). Specifically, grip strength declines observed in TR-HF rats, compared to NC (p<0.01), were attenuated in TR-HF+IBU rats. Likewise, grip strength declines observed in 6-week HRHF rats, compared to NC rats (p<0.001), were attenuated in 6-week HRHF+IBU rats (p<0.05 compared to 6-week HRHF). The 6-week HRHF+IBU rats had grip strengths similar to NC and NC+IBU rats (Fig. 1D).

### Exposure-dependent increases in CTGF and collagen type 1; CTGF increases more with HRHF

**Figure 2:**
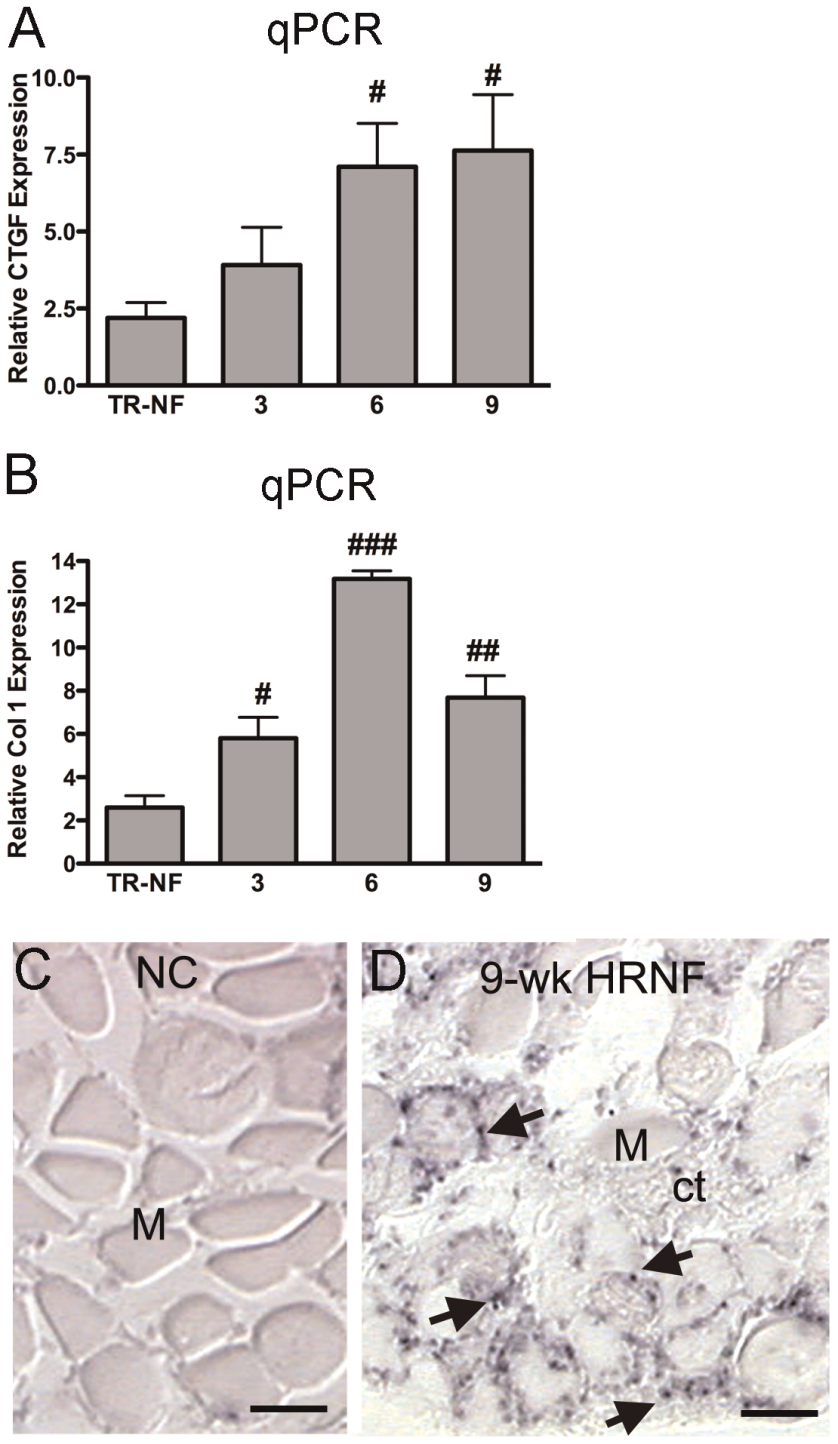
Examination of expression levels of connective tissue growth factor (CTGF) and collagen 1 (Col1) with HRNF task using quantitative PCR (qPCR). RNA was extracted from region A of the flexor digitorum muscles of normal controls (NC), trained-only rats (TR-NF), and HRNF rats that had performed the task for 3, 6 or 9 weeks. *(A,B)* Quantitative real-time PCR (qPCR) showing CTGF and Col1 mRNA expression levels, relative to GAPDH. qPCR data represents average of n = 3/gp. ^#^p<0.05, ^##^p<0.01, ^###^p<0.001, compared to TR-NF. *(C)* NC muscle (M) cut in cross section showed no expression of CTGF mRNA using in situ hybridization. *(D)* 9-week HRNF muscle showed increased CTGF mRNA using in situ hybridization in small cells around myofibers (M) and in surrounding connective tissue (ct). Similar in situ hybridization results were replicated in n = 3/gp. Scale bars = 50 µm.

We initially explored if there were changes in expression of CTGF and collagen type I in rats performing the HRNF task, and found that each increased with continued HRNF task performance (Fig. 2A–D). Using qPCR analyses, we observed a significant upregulation of CTGF expression in 6- and 9-week HRNF, compared to TR-NF rats (Fig. 2A, p<0.01 each) and a significant upregulation of Col1 expression in 3-, 6- and 9-week HRNF, compared to TR-NF rats (Fig. 2B; p<0.05, p<0.001, and p<0.01, respectively). In situ hybridization was used to confirm the CTGF gene expression and showed increased CTGF in 9-week HRNF (Fig. 2D; note small dark stained cells on periphery of myofibers and in surrounding connective tissues), compared to NC muscles (Fig. 2C).

**Figure 3:**
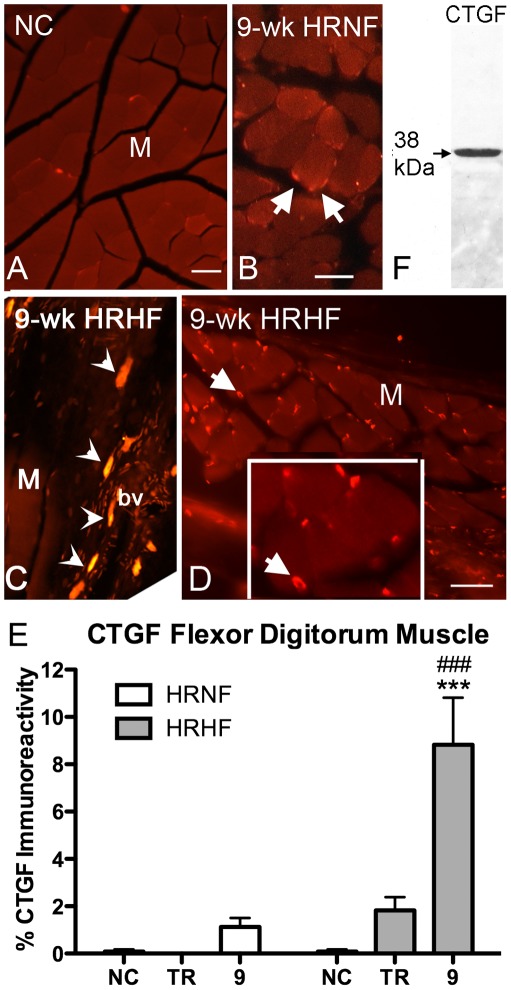
Connective tissue growth factor immunostaining increased more in HRHF muscles than in HRNF. Cross-sections (panels A,B,D), and longitudinal sections (panel C) from region A of flexor digitorum muscles (M) are shown. *(A)* NC muscle showing only low levels of CTGF immunostaining. *(B)* 9-week HRNF muscle showing a small increase in CTGF-immunostained cells at peripheral edges of myofibers (arrows). *(C)* 9-week HRHF muscle showing increased CTGF in larger mast-like cells (arrowheads) near a blood vessel (bv). *(D)* 9-week HRHF muscle also contained smaller CTGF-immunostained cells (arrows) at the periphery of myofibers. Inset shows higher power of the CTGF-immunostained cells at edges of myofibers. *(E)* Quantification of CTGF immunostaining in flexor digitorum muscles. *(F)* A lane from a representative Western blot showing that the CTGF antibody used for the immunohistochemistry detects a band at 38 kDa in the flexor digitorum muscles, the expected molecular weight of CTGF. The whitish band below the 38 kDa band indicates the site of GAPDH. The gel was first stained with anti-GAPDH and then stripped prior to staining with anti-CTGF. ***p<0.001 and ^###^p<0.001, compared to NC and TR-HF, respectively (n = 3–10/gp). Scale bars = 50 µm.

Immuohistochemistry was used to determine if CTGF protein levels also increased, as well as the cellular and spatial location of the CTGF protein. HRNF flexor digitorum muscles showed only a small but non-significant increase in CTGF immunostaining in 9-week HRNF, compared to NC rats (Fig. 3B versus Fig. 3A; Fig. 3E). Therefore, we extended our study to include a higher force demand task (i.e. the HRHF task), and found increased CTGF in large mast-like cells near blood vessels (arrowheads in Fig. 3C), and in many small cells at edges of myofibers (Fig. 3D). Quantification of the immunostaining showed increased CTGF immunoreactivity in 9-week HRHF muscles, compared to NC and compared to 9-week HRNF rats (p<0.001 each; Fig. 3E). Figure 3F shows that the anti-CTGF antibody detected a 38 kDa protein, the expected molecular weight of CTGF, in a western blot of flexor digitorum skeletal muscle from a HRHF animal.

**Figure 4:**
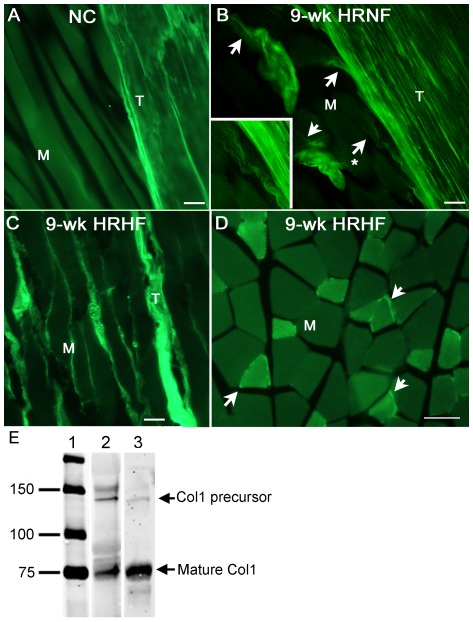
Collagen immunostaining increases in flexor digitorum muscles with HRNF and HRHF tasks. Cross-sections (panels A,B,D), and longitudinal sections (panel C) from region A of flexor digitorum muscle (M) are shown. *(A)* NC muscle (M) showing no immunostaining for collagen type 1 in or around individual myofibers. *(B)* 9-week HRNF muscle showing increased collagen type I staining around a few myofibers. Arrows indicate collagen immunoreactive staining extending around individual myofibers. Inset shown higher power photo of area in panel B indicated with an asterisk. *(C,D)* 9-week HRHF muscle showing increased collagen type I staining in the endomyseum (panel C), and in small cells at the edges of some myofibers (arrows; panel D), as well as within some myofibers (panel D).The T in panel C indicates a small tendon slip within the muscle mass region. Similar results were observed in n = 3/gp. *(E)* Lanes from a representative Western blot of flexor digitorum muscles from HRHF rats probed with the collagen type 1 antibody used for the immunohistochemistry. Lane 1 shows the standards; Lane 2 shows bands at the expected molecular weights of procollagen type I (approximately 140 kDa; [45], [46]) and mature collagen (75 Kda here; known to be between 70 and 90 kDa; [45]); Lane 3 from a different muscle sample showing mainly detection of a band at the molecular weight of mature collagen. Scale bars = 50 µm.

qPCR findings for collagen type I were confirmed qualitatively using immunohistochemistry (Fig. 4). There was no collagen type I immunostaining in or around NC myofibers (Fig. 4A). However, collagen type I immunostaining was visible around myofiber slips at sites where muscles attached to tendons in 9-week HRNF rats (Fig. 4B and inset). Collagen type I immunostaining was even more pronounced in flexor digitorum muscle of 9-week HRHF rats in the endomyseum (Fig. 4C), small cells around myofibers, and within some individual myofibers (Fig. 4D). Figure 4E shows that the anti-collagen 1 type antibody detected bands that matched the expected molecular weights of collagen type 1 precursors and mature collagen type I, in western blots of flexor digitorum skeletal muscles from HRHF rats, matching previously published molecular weights for collagen type 1 [45], [46].

### TGFB1 increases are greater with higher force training and task performance

**Figure 5:**
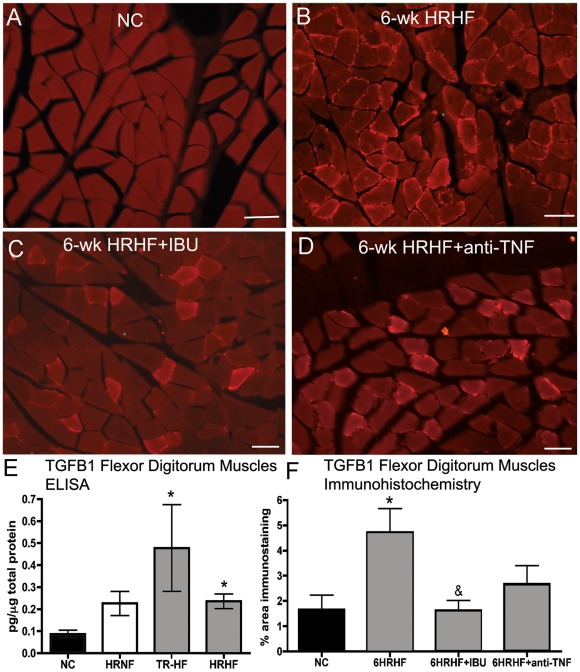
TGFB1 increases in TR-HF and 6-week HRHF rats and is attenuated by ibuprofen. *(* ***A*** *)* NC muscle cut in cross-section showing no TGFB1 immunoreactive cells. *(B)* 6-week HRHF muscle showing TGFB1-immunostained cells at edges of myofibers (arrows). *(C)* Muscle from a 6-week HRHF rat treated with ibuprofen (6-wk HRHF+IBU) showing fewer TGFB1-immunostained cells than in panel B. *(D)* Muscle from a 6-week HRHF rat treated with anti-TNF-α (6-wk HRHF+anti-TNF) showing reduced TGFB1 immunostaining. *(E)* ELISA results for TGFB1 in muscles from NC, 6-week HRNF, TR-HF, and 6-week HRHF rats; n = 3–8/gp. *(F)* Quantification of percent area of muscle with TGFB1 immunostaining. *p<0.05 compared to NC; ^&^p<0.05 compared to untreated 6-week HRHF; n = 4–10/gp. Scale bars = 50 µm.

Since TGFB1 has been shown to be an upstream modulator of CTGF and collagen production in myofibroblasts [32], [41], and has been implicated in muscle fibrosis [41], we looked for its increase in the flexor digitorum muscles (Fig. 5). Immunohistochemistry showed little to no TGFB1 immunostaining in NC rats (Fig. 5A). In contrast, a clear increase of small cells immunopositive for TGFB1 were observed at the periphery of myofibers and in the endomyseum between myofibers in 6-week HRHF rats (Fig. 5B). ELISA was used for quantification of TGFB1 protein levels and revealed significant increases of TGFB1 in TR-HF and 6-week HRHF flexor digitorum muscles, compared to NC rats (p<0.05 each; Fig. 5E). A two-week treatment of HRHF rats with ibuprofen treatment reduced TGFB1 immunoexpression, compared to untreated 6-week HRHF rats (p<0.05; compare Fig. 5C to 5B; Fig. 5F). A two week treatment of HRHF rats with anti-TNF-α treatment also reduced TGFB1 immunoexpression, although not significantly (compare Fig. 5D to 5B; Fig. 5F). TGFB1 levels in 6HRHF+antiTNF and 6HRHF+IBU were not significantly different from NC levels (Fig. 5F).

### Anti-inflammatory drugs decrease CTGF and matrix in HRHF rats

**Figure 6:**
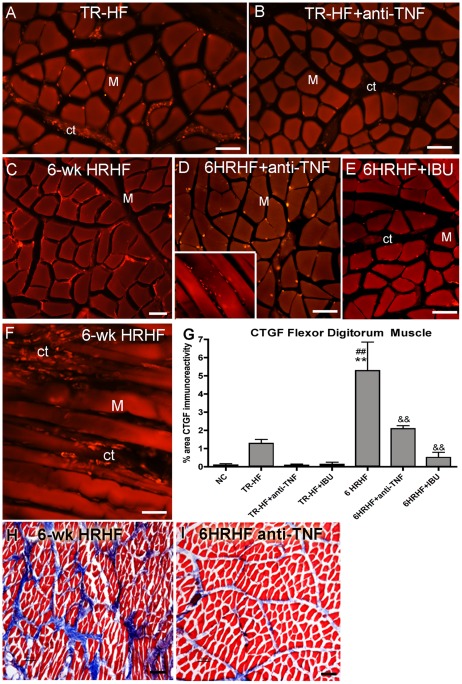
Increased CTGF and collagen staining decreases after anti-inflammatory drug treatments. CTGF immunohistochemistry (panels A–F) and Masson's trichrome staining (panels G,H) increased in flexor digitorum muscles (M) and lumbricals in TR-HF and 6-week HRHF task rats, and decrease with anti-inflammatory drugs. Panels A–F show CTGF immunostaining (red) of flexor digitorum muscles from: *(A)* TR-HF, *(B)* TR-HF treated with anti-TNF-α, *(C)* untreated 6-week HRHF (showing CTGF-immunostained cells at edges of myofibers), *(D)* 6-week HRHF treated with anti-TNF-α (inset shows small CTGF-immunostained cells in endomyseum), *(E)* 6-week HRHF treated with ibuprofen, and *(F)* untreated 6-week HRHF (showing increased CTGF-immunostained cells in endomyseum (ct)). *(G)* Quantification of percent area with CTGF immunostaining. **p<0.01 compared to NC; ^##^ p<0.01 compared to TR-HF; ^&&^p<0.01 compared to untreated 6-week HRHF; n = 4–12/gp. *(F)* Lumbricals from Region B stained with Masson's Trichrome showing: *(H)* increased blue stained collagen matrix in an untreated 6-week HRHF rat, and *(I)* decreased blue staining in a 6-week HRHF treated with anti-TNF-α. Scale bars = 50 µm.

Since production of CTGF by fibroblasts is also regulated by TNF-α [37], [38], we examined the effects of both anti-TNF-α and ibuprofen on CTGF and matrix deposition in the flexor digitorum muscles. Both treatments decreased the initial HRHF-training and HRHF-task induced increases in CTGF and collagen matrix (Fig. 6). TR-HF rats had small increases in CTGF immunoreactivity (Fig. 6A), but little staining was seen in TR-HF-anti-TNF rats (Fig. 6B), although neither change was significantly different from NC (Fig. 6G). The amount of CTGF in TR-HF+IBU was also not significantly difference from that in NC (Fig. 6G). In contrast, CTGF immunoreactivity was greater in untreated 6-week HRHF muscles, compared to NC rats (Fig. 6C,F,G; p<0.01). However, CTGF staining was significantly lower in 6-week HRHF rats treated with either anti-TNF-α or ibuprofen (6HRHF+anti-TNF and 6HRHF+IBU rats), compared to untreated 6-week HRHF rats (Fig. 6D–G; p<0.01 each). Lumbrical muscles of the palm also showed decreased collagen matrix staining in 6HRHF+anti-TNF rats compared to 6-week HRHF rats (compare Fig. 6I to 6H). Similar decreases in collagen matrix staining were observed lumbricals of 6HRHF+IBU rats (data not shown).

## Discussion

Our aims were to determine: a) if fibrogenic-related proteins increase in skeletal muscles in response to prolonged repetitive reaching, b) if their increases parallel declines in grip strength, c) the extent to which increased force loads contribute to either change, and d) if anti-inflammatory treatments attenuate these responses. We found that CTGF and TGFbeta1 increased in flexor digitorum muscles, particularly in cells in the interstitial matrices (endomyseum), in an exposure-dependent manner with greater increases with continued task performance and with higher force. These changes were associated temporally with increased collagen expression and deposition, and grip strength declines. Both anti-inflammatory treatments lead to decreased CTGF and collagen matrix deposition in HRHF rats, despite continued task performance. In contrast, TGFB1 decreased significantly with ibuprofen treatment, but not with anti-TNF-α. Task-induced grip strength declines were also attenuated by ibuprofen treatment, again, despite continued task performance, matching our past results showing improved grip strength with anti-TNF-α treatment [12]. Since both anti-inflammatory drugs attenuated CTGF and collagen production, as well as grip strength declines, it is possible that inflammation-induced matrix changes contribute to declines in grip strength.

Daily exposure (mean reaches/day) was fairly similar in both the food retrieval task and handle-pulling task (the HRNF and HRHF tasks, respectively). Daily exposure was higher than target levels initially in both task groups, and then declined in each group to target levels with continued task production. Thus, one key difference between the tasks was force level (<5% maximum pulling force in the HRNF task versus 60% in the HRHF task).

The progressive declines in grip strength with both tasks supports an exposure dependent influence on this motor variable. The greater declines with HRHF support a force level influence, while the improvement after ibuprofen treatment supports a contribution from inflammatory mechanisms. Since there were greater increases in matrix protein production and interstitial matrices in HRHF than HRNF rats, we postulate that task-induced matrix changes also contributed to the declines in grip strength. This matches results by Cutlip et al showing increased cellular interstitial responses in skeletal muscles after chronic administration of stretch-shortening cycles, and decreased functional performance (decreased isometric and peak eccentric forces), even in the absence of myofiber necrosis [22]. On the other hand, increased muscle inflammatory cytokines, including TNF-α, also correlates with declines in grip strength [6], [16], [47]. We have reported that both systemic ibuprofen and anti-TNF-α treatment attenuates HRHF task-induced increases of inflammatory cytokines in musculoskeletal tissues [12], [42], and that the anti-TNF-α treatment improves grip strength [12]. Furthermore, treatment of subjects with subcutaneous TNF-α receptor before unaccustomed exercise improves muscle strength [48], as does ibuprofen [49], [50]. Thus, we are unable to separate the effects of increased inflammation from increased matrix deposition on grip strength in our model. We can only postulate that our findings combined with those by other groups [22], [48], [49], [50] indicate that both muscle fibrosis and inflammation contribute to our observed repetitive task induced grip strength declines. The extent to which the combined effects of these two processes are additive, or worse, is unknown but raises the possibility that individuals prone to fibrotic matrix deposition may be more susceptible to functional loss with repetitive task exposure than those who experience a predominantly inflammatory response.

Several studies have examined the effects of cyclical loading and repeated muscle strains on extracellular matrix content, including collagen [7], [8], [17], [22], [24], [25], [26], [27], [28], [29]. There is also data concerning the influence of physical exercise, mechanical loading andcyclical loading on CTGF and TGFB1 production in musculoskeletal tissues. We have reported increased CTGF as well as collagen production in involved nerves and tendons of rats performing HRNF and HRHF tasks for 9–12 weeks [7], [8], [17]. Cyclical loading of tendons also results in increased CTGF [30]. Acute exercise does not alter TGFB1 mRNA levels in skeletal muscles [51]. In contrast, prolonged treadmill running of 60 min/day for 6 weeks leads to increased TGFB1 mRNA but not increased TGFB1 protein levels [51], perhaps because their training protocol was geared more towards endurance training rather than overload or overuse. TGFB1, CTGF and collagen type 1 increase in skeletal muscle after 4 days of isometric and eccentric training, although not after concentric training [33]. This suggests that muscle is sensitive to differences in contraction type or force production, although the latter study was not designed to answer that question specifically. A strong link between mechanical loading and TGFB1 mRNA and protein levels in tendons and isolated fibroblasts has been established [32], [39], [52]. Our results now establish a similar link between increased force loads and increased TGFB1, CTGF and collagen type I in skeletal muscles during repetitive task performance using an *in vivo* model.

Our observed fibrotic muscle responses paralleled increases in CTGF and TGFB1. Several groups have shown that CTGF is a secreted matricellular protein associated with tissue repair and fibrosis [41], [52], [53] (also reviewed by [40], [53]. TGFB1 is also an established and potent stimulator of connective tissue formation in skeletal muscle healing after injury, particularly at sites of mechanical stress [32], [33]. CTGF's mediation of collagen production and fibrosis is regulated at least partially by TGFB1 [32], [39], [53], [54]. This explains our findings that TGFB1 increases significantly in TR-HF rat muscles prior to increases of CTGF in HRHF muscles. We also observed that ibuprofen treatment reduced TGFB1 production, despite continued muscle loading, as well as CTGF and collagen matrix. The reduction in TGFB1 by ibuprofen likely contributed to the reduced CTGF; and reduced CTGF contributed to the reduced collage n production and deposition in the muscles.

Mechanical loading of tissues also increases production of fibroblast growth factor (FGF), platelet derived growth factor (PDGF), and vascular endothelial growth factor (VEGF) in muscle and tendon fibroblasts [30], [55], [56]. For example, mechanical loading increases FGF release in vitro from skeletal muscle cells; associated muscle cell growth is inhibited after administration of an antibody that neutralizes FGF activity [57]. FGF2 (basic FGF), and to a lesser extent FGF1 (acidic FGF), are potent stimulators of fibroblast proliferation, collagen formation, and fibrogenic tissues changes [58]. FGF subtypes (FGF-2 and FGF-6) combined with TGFB contribute to collagen production, albeit this has been shown only in chondrocytes to date [59]. These findings indicate that we should examine VEGF, PDGF and FGF subtypes in future studies in our model.

CTGF production also appears to be regulated by TNF-α [37], [38], matching our observation of decreased CTGF and collagen matrix after anti-TNF-α treatment. A direct role for CTGF in tissue fibrosis is controversial, since studies show that a cooperative interaction between CTGF and TGFB is needed for overt tissue fibrosis [32], [39], [53]. On the other hand, Sonnylal and colleagues have reported that overexpression of CTGF by fibroblasts is directly linked to tissue fibrosis in vivo [41]. We have shown that anti-TNF-α treatment-down regulates a related matricellular protein, PLF (periostin like factor) [12]. However, in that study, we found that anti-TNF-α treatment did not reduce CTGF in HRHF rat muscles using western blot analysis. Perhaps homogenization and then Western blot analysis of a large muscle mass is not sensitive enough to detect the early CTGF changes observed here using immunohistochemistry. Concerning the ibuprofen induced decreases in CTGF, we reported recently that ibuprofen treatment reduces task-induced increases of several inflammatory cytokines, including TNF-α, in skeletal tissues in our model [42]. Based on these results combined, we postulate that ibuprofen-induced reductions in tissue inflammatory cytokines and TGFB1 lead to the observed reductions in CTGF in the ibuprofen treated rats.

With regard to which anti-inflammatory drug might be more efficacious for patients with WMSDs, both therapies used in this study seems to decrease the detrimental effect of muscle fibrosis at the expense of CTGF production. Therefore, since Ibuprofen treatment is the least expensive method, it might be the most appropriate therapy. However, its use should be limited to short-term treatments. Ibuprofen medication has been shown in a few studies to suppress protein synthesis after eccentric exercise. It may also inhibit skeletal muscle hypertrophy and adaptation with training [49], [60], [61], [62], although a more recent study shows no effect of ibuprofen on muscle hypertrophy [50]. The usefulness of long-term anti-TNF-α treatments for WMSDS has yet to be explored, although it has been shown to reduce inflammation and scarring in experimental glomerulonephritis and hepatic fibrosis [35], [36]. It is clear though from this study, that despite a continued increase of TGFB1 in HRHF muscles, that CTGF can be targeted to reduce prolonged loading induced skeletal muscle fibrosis. Perhaps even more specific drugs can be developed that are effective, inexpensive and easy to administer to lower CTGF in muscle and therefore fibrosis.

Our results are similar to those by Lapoint in 2002, in an animal model of exercise-induced muscle damage [63]. They found that administration of an anti-inflammatory drug (diclofenac sodium) during the acute inflammation phase (during the first 3 days) after unaccustomed eccentric contractions was effective at restoring muscle force. However, delayed administration of diclofenac sodium to a time point when the inflammatory response was declining had no effect on muscle force. In this study of cumulative repetitive loading across many weeks, the timing of anti-inflammatory drug administration to HRHF rats was at the onset of the inflammatory process. We have previously shown that the macrophage and inflammatory cytokine response begins around week 3 and continues through week 12 in musculotendinous tissues of HRHF rats [12], [17]. We timed the drug administration in this study to be during this early inflammatory phase in the hopes of preventing inflammation-induced catabolic responses. We have yet to investigate the effectiveness of drugs at later time points, but hypothesize that anti-inflammatory drugs will not be effective post inflammation. It is clear from this study that despite a continued increase of TGF-beta 1 in HRHF muscles, CTGF can be targeted more specifically to reduce prolonged loading-induced skeletal muscle fibrosis. There is recent evidence in the literature demonstrating that it is possible to specifically target CTGF to abrogate CTGF-dependent skeletal muscle dystrophy/fibrosis and tumor growth [64], [65]. Levels of CTGF have been shown to be markedly elevated in various injured tissues that develop fibrosis including the skin, kidney, liver and lung [66], [67], [68], [69], [70]. More recently, CTGF over-expression has been implicated in the pathophysiology of dystrophic skeletal muscles where it is believed to contribute to the deterioration of skeletal muscles and their function in addition to mediating the ensuing fibrosis of the damaged muscle tissue [65]. Clinical trials are currently underway using a fully human IgG_1κ_ monoclonal antibody that recognizes domain 2 of human and rodent CTGF (this monoclonal is called FG-3019 and was developed by Fibrogen, Inc., South San Francisco, CA) as a novel therapy to treat patients with Pancreatic cancer, idiopathic pulmonary fibrosis, and liver fibrosis due to chronic hepatitis B infection [71]. This antibody has also been used to treat CTGF-expressing tumors in mice, where it abrogated CTGF-dependent pancreatic tumor growth and lymph node metastasis without any toxic side effects in mice [64]. FG-3019 has also been used as a therapy in a mouse model of Duchenne muscular dystrophy, where it reversed the fibrosis of muscular tissue and even allowed return of skeletal muscle function (personal communication, Dr. Enrique Brandan). These studies support the concept of using drugs that specifically target CTGF as a treatment to prevent reduced skeletal muscle function and the ensuing fibrosis that occurs as a consequence of overuse in WMSDs.

It is also possible that genetic predisposition to fibrosis may, in part, explain why some individuals are more prone to functional losses with exposure to repetitive tasks. Models that explore the interactions between such genetic factors and environmental exposures may provide additional insight into and guidance toward appropriate and individualized treatment approaches.

In conclusion, these data suggest that continuous repetitive loading of skeletal muscles, particularly with high force, stimulated the production of fibrogenic proteins (CTGF and TGFB1). This increase was accompanied by increased collagen synthesis and matrix deposition as well as reduced grip strength. Early treatment with anti-inflammatory drugs (anti-TNF-α and ibuprofen) successfully attenuated production of CTGF and collagen matrix deposition. Ibuprofen treatment also attenuated TGFB1 production. Lastly, grip strength improved with both anti-inflammatory treatments. However, since this is a short study, with only short-term anti-inflammatory treatments, similar anti-fibrotic results may not be sustained with longer task regimens.
